# Physical and Chemical Characterization, Adsorption Kinetics, Thermodynamic Analysis, and the Mechanism Involved in the Removal of Methylene Blue Dye by a Biosorbent from Pecan Nutshells

**DOI:** 10.1021/acsomega.5c12140

**Published:** 2026-01-22

**Authors:** Lucas M. Frescura, Rogerio V. Lourega, Nicole W. da Silva, Marcelo B. da Rosa

**Affiliations:** † Post-Graduate Program of Pharmaceutical Science, 28118 Universidade Federal de Santa Maria, Avenue Roraima n 1000, Santa Maria 97000-001, Rio Grande do Sul, Brazil; ‡ Chemistry Departament, Federal University of Santa Maria, Avenue Roraima n 1000, Santa Maria 97000-001, Rio Grande do Sul, Brazil

## Abstract

The search for low-cost and sustainable adsorbents for dye removal has driven the valorization of agro-industrial residues. In this study, pecan nutshells (PNSs) were evaluated as a biosorbent for methylene blue (MB) removal. The material was prepared by drying, grinding, and alkaline treatment with NaOH, which led to an increase in the apparent surface area (0.974 m^2^·g^–1^) and pore volume (0.0014 cm^3^·g^–1^). Surface characterization revealed the involvement of hydroxyl, carboxyl, and aromatic functional groups in the interaction with the dye molecules. The adsorption of MB by PNSs exhibited high efficiency, even at low dosages, and optimal performance was observed at 1.5 g·L^–1^. The process remained essentially pH-independent across the 4–10 range. Equilibrium data were analyzed using the Langmuir, Freundlich, Dubinin–Radushkevich, and Sips isotherm models. Among them, the Sips model provided the best fit to the experimental data, yielding a maximum adsorption capacity of 317.5 mg·g^–1^ at 25 °C, which is consistent with a heterogeneous surface presenting finite saturation. The thermodynamic parameters (Δ*H*° = +12.32 kJ·mol^–1^; Δ*G*° = −13.43 kJ·mol^–1^ at 25 °C; Δ*S*° = +86.27 J·mol^–1^·K^–1^) indicate that the adsorption process is spontaneous and endothermic, with an enthalpy change characteristic of weak to moderate interactions. Kinetic data were best described by the pseudo-second-order model, with equilibrium reached within approximately 180 min. Intraparticle diffusion analysis revealed a multistep adsorption involving an initial boundary-layer diffusion followed by a slower pore diffusion. Overall, the adsorption mechanism is interpreted as multifactorial, governed by hydrogen bonding, π–π stacking, and van der Waals forces, consistent with a physisorption-dominated process strengthened by structural heterogeneity. These results demonstrate that PNS combines high capacity with operational simplicity, representing a scalable and sustainable alternative for the treatment of dye-contaminated wastewater.

## Graphical abstract

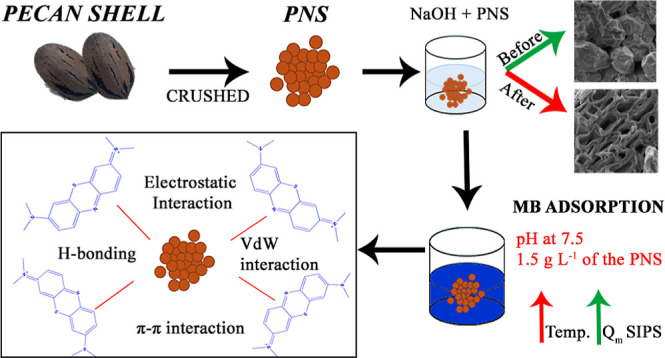

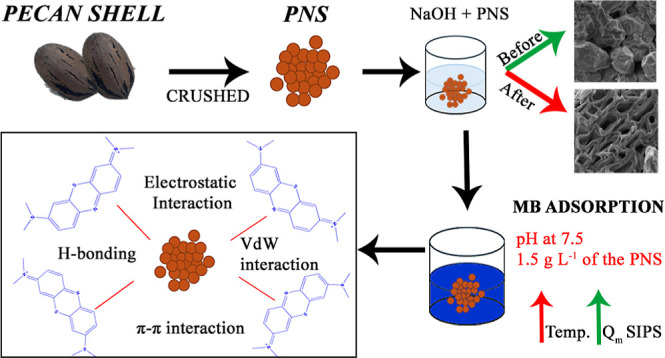

## 1 Introduction

The textile sector plays a significant role in the global economy owing to its large-scale production. However, the volume of waste (liquid, solid, and gaseous) generated by this sector raises environmental concerns.^1^ One of the main issues is the removal of synthetic dyes from wastewater, as the textile industry not only leads the usage of dyes but also produces approximately 100 tons per year of this type of effluent.^2^ Furthermore, the textile sector is one of the largest consumers of water, generating 50 to 100 L of effluent per kilogram of fabric produced, which is often contaminated with organic pollutants and dyes, thereby significantly impacting the environment and human health.^3,4^

Among the most widely used dyes are those characterized by one or more −N=N– groups, which are commonly linked to aromatic systems. These dyes exhibit structural features that confer greater chemical stability.^5^ This class includes methylene blue (MB), a cationic dye widely used for dyeing cotton and wool fabrics. MB remains stable in an aqueous medium and has strong adsorption properties on solid supports.^6^ However, when not properly treated, its release into rivers and lakes affects water transparency, limits the passage of solar radiation, and reduces natural photosynthetic activity, leading to changes in aquatic biota as well as acute and chronic toxicity in these ecosystems.^7,8^ Thus, due to its extensive application, it is necessary to pretreat wastewater containing this dye before releasing it into bodies of water. Among the new and varied technologies studied to minimize such risks,^9^ the use of biomass-derived waste is particularly notable.

Several methods involving physical, chemical, and biological processes have been employed to treat dye-containing wastewater, such as coagulation, membrane separation, advanced oxidation processes (AOPs), and biological treatment.^10,11^ These techniques can be effective under specific conditions, but they often require high operational costs and significant energy input and generate secondary waste streams that demand additional treatment steps and complex process control.^12^ Recent studies have highlighted adsorption as a simple, flexible, and cost-effective process with high removal efficiency, specificity, and a nonpolluting characteristic. The use of renewable and waste-derived materials also contributes to making adsorption one of the most attractive technologies for dye removal.^13,14^

MB is frequently utilized as a model compound in studies on the removal of dyes and organic contaminants from aqueous solutions due to its cationic structure, chemical stability, high solubility, and strong interaction with negatively charged or oxygen-containing functional groups present in lignocellulosic biomasses.^15^ In recent years, adsorbents derived from agricultural byproducts have been used as an economical and realistic method for the removal of dyes from wastewater. Among these biosorbents, those derived from biomass waste stand out, such as lemongrass leaves,^16,17^ banana peels,^18^ *Citrullus colocynthis* seeds and peels,^19^ rice husk,^20^ torrefied rice husk,^21^ acacia wood-based activated carbon (AWAC),^22^ Tucumã seeds,^23^ pecan nutshell activated,^24^ *Dacryodes edulis* leaf,^25^ and watermelon seed hulls.^26^

Agricultural wastes are available on a large scale, are renewable, and often require minimal processing (washing, drying, and grinding), thus reducing costs compared to other adsorbent materials. Furthermore, the presence of surface functional groups, such as the carboxyl group, in agricultural waste has been demonstrated to enhance the adsorption capacity of cationic dyes.^27^

Brazil has vast biomass diversity and industrializes a wide range of products, consequently generating significant amounts of waste. The valorization of these materials aligns with modern biorefinery strategies, in which agroindustrial byproducts are fractionated or chemically modified to produce materials with tailored functionalities for environmental applications. Thus, among the various biomass residues, pecan biomass exhibits a significant potential for biosorption applications. Rio Grande do Sul is the main pecan-producing state in Brazil, showing a significant increase in the planted area and production over recent years. According to the IBGE (Brazilian Institute of Geography and Statistics), the state accounts for 92% of the cultivated area and 88% of total pecan production in the country. In the 2023/2024 harvest, for example, it reached 3200 tons from a total area of 7120 ha, considering that approximately 50% of the total fruit weight corresponds to pecan residues, mainly the shell.^28^

Pecan nut waste, such as shells and the cake resulting from oil processing, can be utilized in a variety of applications, including animal feed, residue meal production, oil and tannin extraction, and activated charcoal production. Furthermore, pecan nut wastes are rich in antioxidants and can be used in teas and as ingredients in culinary preparations.^29^ The use of pecan nut waste contributes to reducing the environmental impact of industry while simultaneously generating value-added products.

This work aims to use pecan nutshells (PNSs) as biosorbents, aiding in the removal of MB dye from industrial wastewater. This approach is based on the biorefinery concept, considering sustainability aspects, from material preparation to biosorbent dosage, as well as the influence of the medium’s pH and overall effectiveness. Given the large number of studies on MB adsorption reported in the literature, comparing the performance of different biosorbents remains essential to identifying materials with practical applicability. Therefore, this work also discusses the adsorption capacity of PNSs in comparison to other lignocellulosic adsorbents, providing a comparative assessment supported by recent studies.

## 2 Materials and Methods

### 2.1 Biosorbent Preparation

The pecan shells were obtained as waste from the harvest of a rural property located in the state of Rio Grande do Sul, Brazil (30°18′44.9″ S, 52°45′06.8″ W). The shells were initially ground in a knife mill (Kie, Louveira, Brazil) and washed with a total of 4 L of distilled water in four successive stages. Subsequently, the material was dried in an oven at 40 °C for 72 h. After drying, granulometric separation was performed to obtain particle sizes between 0.25 and 0.42 mm. These particles were treated with an aqueous solution of 0.01 mol L^–1^ sodium hydroxide (NaOH) and washed until the filtrate was colorless. The material was then dried again under the same conditions (40 °C for 72 h) and stored away from light and moisture.

### 2.2 Biosorbent Characterization

The pecan nutshell (PNS) was characterized by Fourier transform infrared spectroscopy (FTIR), performed in a Bruker VERTEX 70v spectrometer equipped with a diamond crystal ATR accessory; the spectral window ranged between 4000 and 400 cm^–1^. The surface area, pore volume, and pore size were determined using the Brunauer–Emmet–Teller (BET), Langmuir, and Barrett, Joyner, and Halenda (BJH) methods, with adsorption/desorption of N_2_ in a Micromeritics analyzer (ASAO, 2020; USA) operating at 77 K. Scanning electron microscopy (SEM) was carried out using a VEGA-3G microscope (Tescan, Czech Republic) equipped with a secondary electron detector to analyze the morphology of the materials. Prior to the analysis, the samples were coated with gold through a sputtering metallization process, applying a current of 30 mA for 120 s using the Desk V system from Denton Vacuum. The point of zero charge (PZC) was determined in 50 mL Erlenmeyer flasks, each containing 25 mL of 0.1 M NaCl solution. The pH of the solutions was adjusted to values ranging from 2 to 12 by adding 1 M HCl or NaOH solutions. The adsorbent dosage was set at 1.5 g L^–1^, and the solutions were maintained under constant agitation for 48 h. This procedure was adapted from Yağmur and Kaya (2021).^30^ Initial and final pH values of the solution were measured using a pH meter (Model One Sense pH 2500, Marte Cientifica).

### 2.3 PNS Quantities and pH Influence Assays

The adsorption of methylene blue (MB) onto PNSs was carried out by using an orbital stirrer (Cientlab) set at 90 rpm. To assess the influence of adsorbent dosage, experiments were conducted with 0.5, 1.0, 1.5, and 2.0 g·L^–1^ of PNS in 50 mL of MB solution at an initial concentration of 500 mg·L^–1^. The influence of pH on adsorption was evaluated at pH values of 4, 6, 8, and 10, adjusted using 1 M HCl or NaOH solutions in an MB solution (500 mg L^–1^) and an adsorbent dosage of 1.5 g·L^–1^. For both assays, the adsorption system was maintained for 20 h to ensure adsorption equilibrium. The adsorption capacity (*q*_e_) was calculated according to eq 1.

1 $$q_{e}=\frac{C_{0}-C_{e}}{m}V$$

### 2.4 Batch Adsorption Assays

Isotherm and thermodynamic studies were performed at temperatures of 5 °C, 15 °C, 25 °C, and 35 °C (±1 °C) in a BOD chamber equipped with digital temperature control (model SSBODu 342L), with the system equilibrated for 20 h. The MB solutions used in these experiments had initial concentrations ranging from 200 to 500 mg·L^–1^ at a pH of 7.5. The residual concentration of MB in the aqueous phase was determined at 661 nm using a PerkinElmer Lambda 16 UV–vis spectrophotometer, and the adsorption data were fitted to the Langmuir (eq 2), Freundlich (eq 3), Dubinin–Radushkevich (eq 4), and Sips (eq 5) isotherm models.

2 $$q_{e}=\frac{\left(q_{\max}K_{L}C_{e}\right)}{\left(1+K_{L}C_{e}\right)}$$

3 $$q_{e}=K_{F}C_{e}^{n}$$

4 $$q_{e}=q_{m}\exp\left(-K{\left(RT\;\ln\left(\frac{C_{s}}{C_{e}}\right)\right)}^{2}\right)$$

5 $$q_{e}=\frac{q_{m}K_{SP}C_{e}^{n_{SP}}}{1+KC_{e}^{n_{SP}}}$$

where *C*_0_ and *C*_e_ (mg L^–1^) are the initial concentration and concentration at equilibrium of MB, respectively; *m* is the mass of PNS; *V* is the volume of the solution; q_e_ (mg·g^–1^) is the amount of MB adsorbed; *q*_max_ (mg g^–1^) is the maximum monolayer adsorption capacity; *K*_L_ (L·mg^–1^) is a constant related to the affinity between the adsorbent and adsorbate; *K*_F_ (mg·g^–1^)/(mg·L^–1^) is the Freundlich constant; *n* (dimensionless) is the Freundlich intensity parameter, which indicates the magnitude of the adsorption driving force or the surface heterogeneity; and *K*_SP_ and *n*_SP_ are the Sips isotherm constant and isotherm exponent, respectively.

The thermodynamic parameters are defined by eqs 6 and 7.

6 $$\Delta G^{{}^{\circ}}=-RT\;\ln K_{C}$$

7 $$\ln K_{C}=-\frac{\Delta H}{R}\frac{1}{T}+\frac{\Delta S}{R}$$

where *R* is the universal gas constant (8.3144 J mol^–1^ K^–1^), *T* is the absolute temperature in Kelvin, Δ*G*° (kJ·mol^–1^) is the Gibbs free energy change, Δ*H*° (kJ·mol^–1^) is the enthalpy change, Δ*S*° (J mol^–1^ K^–1^) is the entropy change, and *K*_c_ is the adsorption equilibrium constant, determined according to eq 8.^31^

8 $$K_{c}=\frac{kMm\gamma^{M}}{\gamma }$$

where *T* is the temperature (K), *R* is the universal gas constant (8.314 J·mol^–1^ K^–1^), *k* is the isotherm equilibrium constant (L·mg^–1^), MM is the molecular weight of methylene blue (g·mol^–1^), γM is the activity coefficient of MB in solution (assuming γM = 1), and γ represents the unit activity coefficient of methylene blue activity (mol·L^–1^).

The adsorption kinetics were evaluated using pseudo-first-order (PFO) and pseudo-second-order (PSO), described by eqs 9 and 10, respectively. The intraparticle diffusion (ID) model is expressed in eq 11.

9 $$q_{t}=q_{e}\left(1-e^{-k_{1t}}\right)$$

10 $$q_{t}=\frac{q_{e}^{2}k_{2}t}{1+k_{2}q_{e}t}$$

11 $$q_{t}=k_{p}t^{1/2}+C$$

where *q*_e_ and *q*_t_ are the amounts of adsorbate uptake per mass of adsorbent at equilibrium and at any time *t* (min), respectively; *k*_1_ (min^–1^) is the rate constant of the PFO model; *k*_2_ (mg·g^–1^ min^–1^) is the rate constant of the PSO model; *k*_p_ (mg·g^–1^ min^–1/2^) is the intraparticle diffusion rate constant; and *C* (mg·g^–1^) is a constant related to the resistance to intraparticle diffusion.

## 3 Results and Discussion

### 3.1 Physical Characteristics of the Biosorbent

The data from the surface analysis of the PNS biosorbent are shown in Table 1. The BET surface area of the material treated with NaOH is higher than that of the sample washed only with water, indicating an increased porosity and exposure of active sites after chemical treatment. This trend was also reflected in the Langmuir surface area values. The external surface area estimated by the t-plot method for the alkalized sample was also higher, confirming the greater exposure of nonmicroporous surfaces, consistent with the negative values of microporous volume observed.^32^ The NaOH treatment also increased the pore volume, which suggests its effectiveness in expanding porous channels or removing pore-blocking obstructions, thereby favoring adsorption. The pore size distributions indicate a predominance of mesopores (2–50 nm), with average diameters between 7 and 8 nm according to the BJH desorption method. The values obtained by BET also reinforce this mesoporosity, with averages close to 5–6 nm.

**Table 1:** Surface Analyses of Carya Nutshells

parameter	water-washed PNS	NaOH-PNS
BET surface area (m^2^·g^–1^)	0.621	0.974
Langmuir surface area (m^2^·g^–1^)	0.969	1.632
BJH adsorption surface area (m^2^·g^–1^)	0.171	0.593
single-point adsorption pore volume(cm^3^·g^–1^)	0.000867	0.001397
t-plot micropore volume (cm^3^·g^–1^)	–0.000140	–0.000324
BJH adsorption pore volume	0.000849	0.00148
adsorption average pore width 9 (nm)	5.59	5.73
BJH adsorption average pore diameter (nm)	19.9	9.99

The point of zero charge (pH_PZC_) of the PNS biosorbent was determined using the pH drift method over an initial pH range of 2 to 12. Figure 1B shows the difference between the final and initial pH (ΔpH) as a function of the initial pH after 48 h of equilibrium.^30^ The point at which ΔpH equals zero corresponds to pH_PZC_, which was estimated to be approximately 6.4. At this pH, the surface of PNS exhibits a net zero surface charge. At pH values below pH_PZC_, protonation of surface functional groups results in a predominantly positively charged surface, favoring the adsorption of anionic species. Conversely, at pH values above 6.4, deprotonation of functional groups leads to a predominance of negative surface charges, enhancing the adsorption of cationic species such as MB.^33^

**Figure 1:**
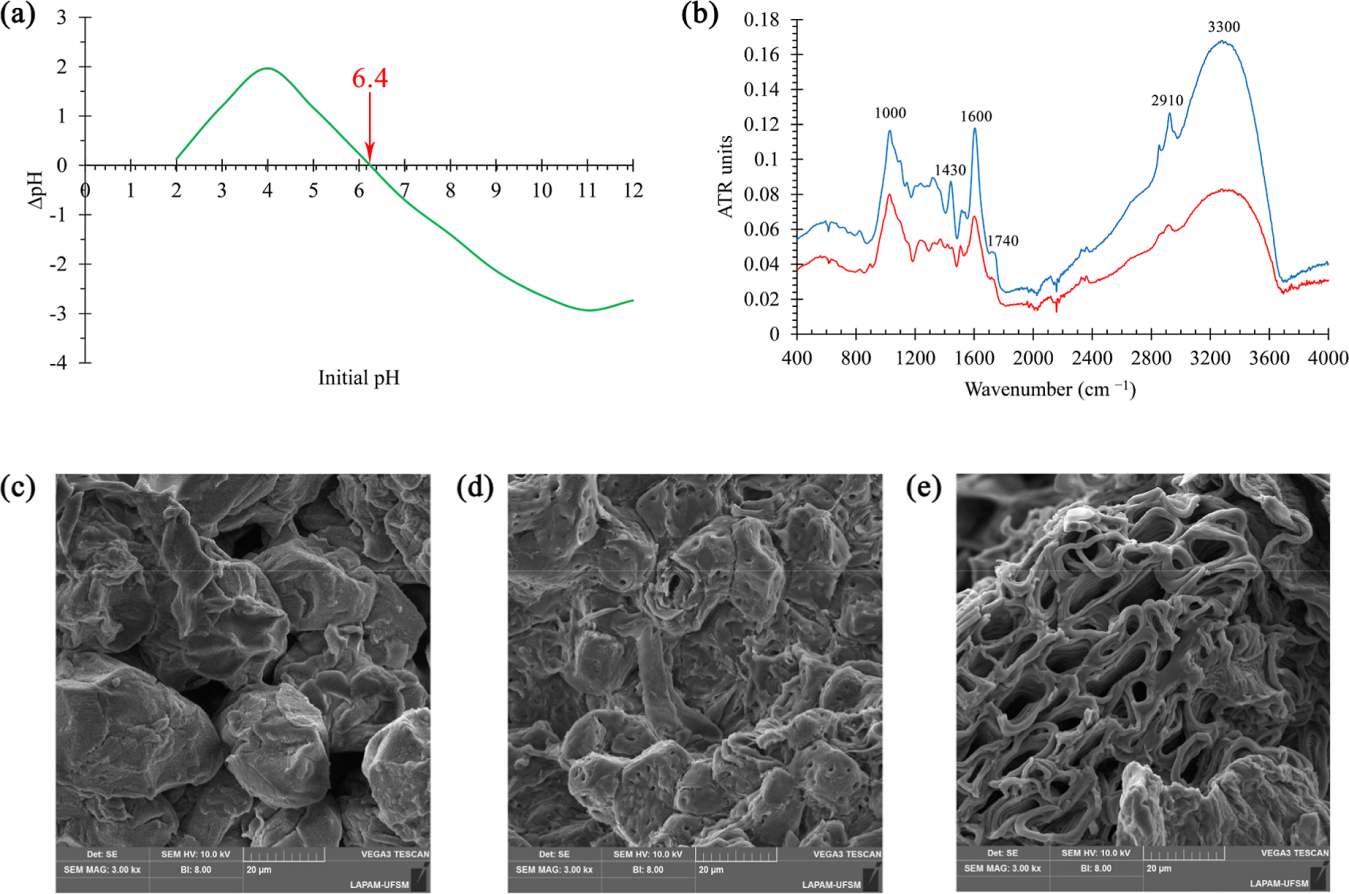
Point of zero charge (PZC) of the PNS biosorbent (a), ATR-FTIR characterization of PNS washed in water (blue line) and treated with NaOH (red line) (b), and SEM image of PNS washed with H_2_O (c) and treated with NaOH in different regions (d,e).

Figure 1a presents the ATR-FTIR vibrational spectra of the PNS (blue line). The pronounced band in the 3000–3600 cm^–1^ region can be attributed to O–H stretching from adsorbed water molecules on the shell, as well as functional groups present in the adsorbent structure, such as phenolic acids. The bands in the 2800–3000 cm^–1^ region are associated with C–H stretching of aliphatic compounds and aldehyde groups. The signal at 1600 cm^–1^ can be attributed to C=O stretching, which is associated with carbonyl-containing functional groups. The band at 1400 cm^–1^ may be related to the phenyl group of the secondary metabolites. Finally, the signals between 1000 and 1300 cm^–1^ are often assigned to C–O bonds characteristic of phenolic compounds.^34^ The red line represents the spectrum of PNS prepared by treatment with a NaOH solution. A decrease in the signal intensity is observed, indicating partial removal or modification of the fibrous structure of the material, which is composed of cellulose, lignin, and hemicellulose. This effect is particularly evident by the absence of peaks at 1430 cm^–1^ and 1740 cm^–1^, as well as the significant reduction of the band at 2910 cm^–1^, attributed to cellulose.^35^

The SEM images illustrate that the alkaline treatment applied to the PNS biosorbent resulted in substantial alterations to the material’s surface morphology. As illustrated in Figure 1d,e, which were acquired from disparate regions of the same sample, there is a marked morphological heterogeneity, a common characteristic of natural lignocellulosic precursors.^36,37^ In specific regions, a more compact and collapsed structure is observed with irregularly distributed and partially obstructed pores, which are associated with more densely lignified areas (Figure 1d). In contrast, other regions exhibit a more open structure, characterized by the formation of cavities and interconnected channels at the micrometric scale as well as clearly defined lamellar walls (Figure 1e). This structural variability indicates that the action of the alkaline agent proceeds in a nonuniform manner, as it is strongly influenced by the anatomical organization of the material and by the local diffusion of the NaOH solution.

Despite this heterogeneity, all analyzed regions of the treated sample exhibited rougher and more porous surfaces than the untreated biomass shown in Figure 1c. This suggests partial removal of amorphous constituents, such as hemicellulose and surface lignin, as well as swelling and reorganization of the lignocellulosic matrix. Such structural modifications have been extensively documented in the existing literature concerning mild alkaline treatments and are associated with the exposure of previously inaccessible functional sites.^38–41^

### 3.2 Mass Dosage and pH Influence in MB Removal

One of the main parameters influencing the removal capacity of an adsorbent material is the adsorbent dosage.^42^ A dosage range of 0.5 to 2 g·L^–1^ of PNS was added to a MB solution with an initial concentration of 500 mg·L^–1^ to investigate the effect of adsorbent dosage. Figure 2A shows the relationship between the adsorption capacity (*q*_e_) and the percentage removal as a function of PNS biosorbent dosage. This relationship demonstrates an inverse correlation between *q*_e_ and biosorbent dosage: higher adsorption capacities are obtained at lower dosages, whereas the percentage of removal increases with increasing dosage. This phenomenon occurs because, at higher dosages, the same amount of solute is distributed across a larger mass of adsorbent. This results in underutilization of adsorption sites and a subsequent decrease in *q*_e_.^43^ Although 2 g L^–1^ resulted in the highest percentage removal (98.6%), its adsorption capacity decreased to 246.6 mg·g^–1^ due to the dilution effect. Conversely, the dosage of 1.5 g L^–1^ represents a more efficient operating point, exhibiting a high removal efficiency (91%) in conjunction with a substantially higher adsorption capacity (303.55 mg·g^–1^ at 25 °C). Consequently, 1.5 g L^–1^ was selected for the subsequent experiments, as it optimizes the adsorbent efficiency per unit mass while maintaining a high overall removal. These results highlight the strong potential of PNS as a low-cost biosorbent. For comparison, Saha (2010) reported a similar removal efficiency using 10 g L^–1^ of tamarind fruit shell, demonstrating the superior performance of PNSs at a substantially lower dosage.^44^

**Figure 2:**
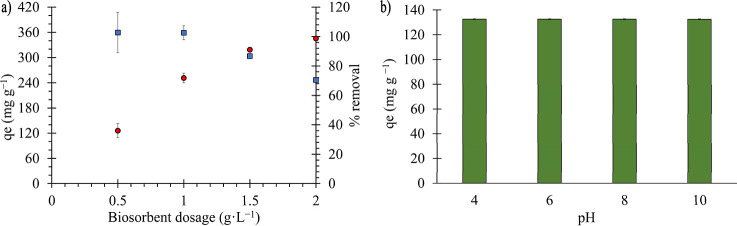
Mass dosage tests with the blue squares representing the adsorption capacity in mg g^–1^ and the red circles representing the removal percentage of MB at an initial concentration of 500 mg·L^–1^ (a) and pH influence assay (b).

The influence of pH on methylene blue adsorption was also investigated over a pH range of 4–10, using solutions with an initial MB concentration of 250 mg L^–1^. As shown in Figure 2B, no significant differences in adsorption capacity *q*_e_ were observed throughout the analyzed pH range. In general, MB adsorption by biosorbents strongly depends on the pH of the system since it influences the charge distribution on the surface of the adsorbent and adsorbate, governing attractive or repulsive electrostatic interactions. MB is a cationic dye with p*K*_a_ values around 3.6 and 11.5. The pH range used in this study is between the p*K*_a_ values of MB, which implies a predominance of the cationic species MB^+^.^33^

The pH_PZC_ value of PNS is 6.4, which indicates that, below this pH, the surface of the adsorbent is predominantly positively charged, disfavoring the electrostatic adsorption of cationic species such as MB. However, the results show that the maximum adsorption capacity is maintained even below the pH_PZC_. This behavior was also observed by Amode et al. (2016), who reported significant differences in adsorption only at a pH value below 2. According to the authors, when the number of available active sites greatly exceeds the number of MB molecules, pH changes in a moderate range do not significantly impact the adsorption efficiency, even if the surface charge of the adsorbent varies.^45^ pH values below 4 and above 10 were not considered in this study, because they do not represent conditions commonly found in natural environments. Thus, the remaining experiments were conducted at pH 7.5.

### 3.3 Adsorption Isotherms and Thermodynamic Analysis

The adsorption equilibrium of methylene blue on the PNS biosorbent was studied using the Dubinin–Radushkevich, Freundlich, Langmuir, and Sips isotherm models at temperatures of 15 °C, 25 °C, 35 °C, and 45 °C (Figure 3a–d). As displayed in Table 2, the experimental data showed a better fit to the Sips isotherm, which combines characteristics of the Langmuir and Freundlich models, describing adsorption on heterogeneous surfaces with saturation behavior.^46^ The satisfactory fit of this model suggests the presence of adsorption sites with different energies and the coexistence of homogeneous and heterogeneous characteristics on the surface of the adsorbent, in accordance with the SEM analysis in Figure 1. Such behavior is common in natural materials used for dye biosorption, including methylene blue.^46–48^

**Figure 3:**
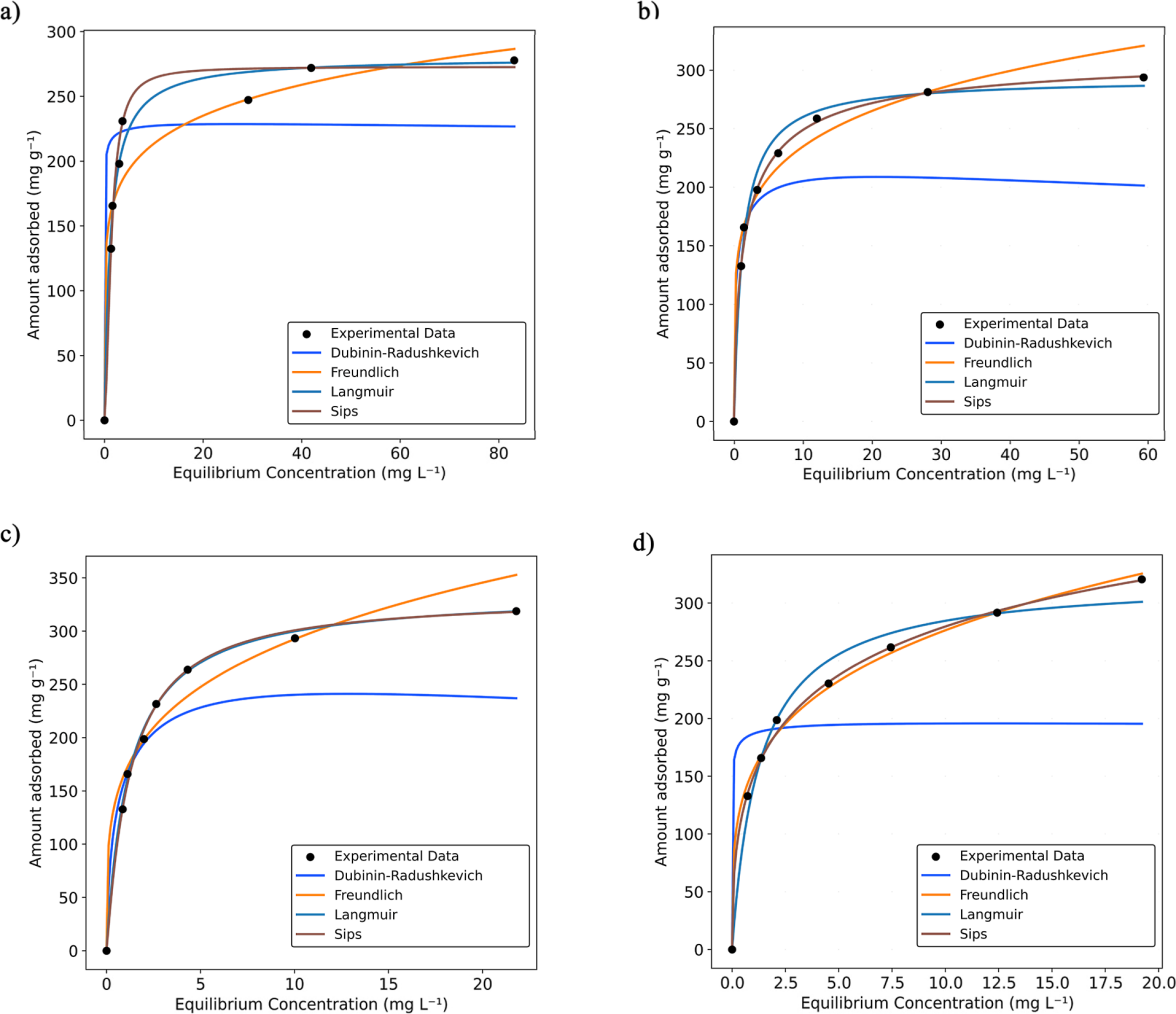
Isotherm equilibrium curves for the biosorption of MB by Carya nutshells at 15 °C (a), 25 °C (b), 35 °C (c), and 45 °C (d).

**Table 2:** Equilibrium Isotherm for the Biosorption of MB onto Carya Nutshells at Different Temperatures

isotherm model	parameters	15 °C	25 °C	35 °C	45 °C
Dubinin–Radushkevich	*q*_m_ (mg·g^–1^)	228.6	208.8	241	195.7
	*K*_DR_	1.08 × 10^–9^	5.30 × 10^–9^	9.39 × 10^–9^	1 × 10^–9^
	*C*_S_	26.58	20.73	1.28	12.2
	RSME	45.5	47.5	39	65.7
Freundlich	*n*	0.139	0.175	0.24	0.25
	kF	154.9	156.7	168	155.4
	RSME	22.1	15.7	19.6	6.49
Langmuir	*q*_m_ (mg·g^–1^)	280.1	292.6	336.3	321
	kL	0.82	0.797	0.827	0.78
	RSME	11.9	9.07	5.28	12.3
Sips	*q*_m_ (mg·g^–1^)	272.9	317.5	332.8	570.67
	*K*_S_	0.579	0.705	0.792	0.942
	*n*_S_	1.71	0.711	1.07	0.431
	RSME	11.3	5.08	5.18	4.05

An increase in temperature resulted in an increase in both the maximum adsorption capacity (*q*_m_), from 272.9 to 570.67 mg g^–1^, and the Sips constant (*K*_Sips_), from 0.579 to 0.942, as the temperature increased from 15 to 45 °C, indicating that increasing the temperature enhances both adsorption capacity and affinity. This behavior is typical for the adsorption of most dyes from solutions and can be explained by factors such as higher MB solubility in solution, increased molecular mobility, a decrease in medium viscosity, and the enhanced diffusion of MB into the pores of the material. In addition, an increase in temperature may cause a slight activation of additional adsorption sites on the surface of the PNS due to a possible expansion of the material, which does not occur at lower temperatures.^49,50^

Thermodynamic analysis of the adsorption process provides essential insight into the nature and mechanisms involved in the interaction between the dye and the surface of the adsorbent. The thermodynamic parameters were obtained from the graph in Figure S1 (one *T* vs ln *K*c). The positive value of the standard enthalpy (Δ*H* = 12.32 kJ·mol^–1^) indicates that the process is endothermic, i.e., it is accompanied by heat absorption. This value is within the typical range of weak to moderate interactions, such as van der Waals (VdW) forces, electrostatic interactions, and weak hydrogen bonds.^51^ This behavior indicates that increasing the temperature favors adsorption and is in line with the results of the experimental adsorption isotherm tests. The endothermic nature of the process can be explained by the breakdown of the strong interactions between the MB and water molecules.^52^ The entry of the dye into the adsorbent matrix implies overcoming solute–solvent interactions, justifying energy consumption. In addition, a higher temperature can cause physical or chemical reorganization of the adsorbent’s surface, promoting the activation of previously inaccessible or low-energy adsorption sites, which can also contribute to enhanced dye retention capacity.^53,54^

The spontaneity of the process is evidenced by the negative values of the Gibbs free energy variation, −12.51 kJ·mol^–1^, −13.43 kJ·mol^–1^, −14.27 kJ·mol^–1^, and −15.1 kJ·mol^–1^ at temperatures of 15 °C, 25 °C, 35 °C, and 45 °C, respectively. Even with a positive enthalpy change, the fact that Δ*G* is negative indicates that entropy is high enough to compensate for heat absorption, leading to a thermodynamically favorable process. In other words, the increase in disorder in the system plays a fundamental role in the viability of adsorption. In fact, the positive values of entropy variation (Δ*S* = 86.27 J·mol^–1^) indicate that the degree of disorder in the system rises throughout the process. This phenomenon can be attributed to the release of water molecules previously organized around the methylene blue molecules or on the surface of the biosorbent itself. When adsorption occurs, these molecules are displaced and released into the aqueous medium, increasing the freedom of movement of the species involved and, consequently, the entropy of the system as a whole.^55^ Another possible explanation for the positive entropy change is the redistribution of the dye molecules along the heterogeneous surface of the shell.

### 3.4 Adsorption Kinetics

Figure 4 shows the fit of the PFO, PSO, and intraparticle diffusion kinetic models for the adsorption of methylene blue onto the PNS biosorbent. The PFO and PSO models are based on the adsorption capacity of the material, whereas the intraparticle diffusion model evaluates whether this diffusion is a limiting step in the adsorption rate.^56^ According to Figure 4a, the nonlinear PSO model fits the experimental data better than the PFO model. This is consistent with the RMSE values presented in Table 3, which also show adsorption capacity (*q*_e_) and kinetic constant (*k*) data for both models. The PSO model further confirms the suitability of PNS as an MB adsorbent material, as this model indicates that the adsorbent has many active adsorption sites and reaches equilibrium within 180 min at an initial working concentration of 250 mg·L^–1^.^57^ The kinetic data are consistent with those of other MB biosorption studies.^23,58^

**Figure 4:**
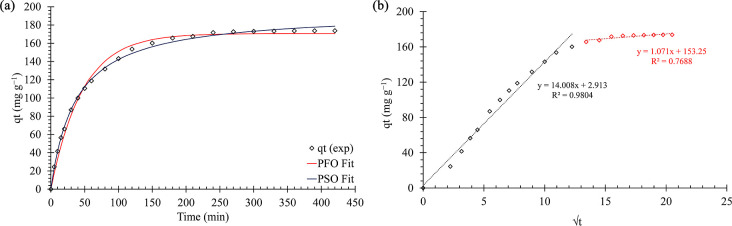
Adsorption kinetics of the pseudo-first-order and pseudo-second-order (a) and intraparticle diffusion kinetics model (b).

**Table 3:** Kinects Parameters Calculated for PFO, PSO, and Intraparticle Diffusion Models

pseudo-first-order		
*q*_e_ (mg·g^–1^)	*k*_1_ (min^–1^)	RSME
170.6782	2.16 × 10^–2^	23.06

pseudo-second-order		
*q*_e_ (mg·g^–1^)	*k*_2_ (mg·g^–1^ min^–1^)	RSME
194.3	1.40 × 10^–4^	12.0

intraparticle diffusion		
*k*_p1_ (mg·g^–1^ min^–1/2^)	*C*_1_ (mg·g^–1^)	*R* ^2^
14.0	2.91	0.9804

intraparticle diffusion		
*k*_p2_ (mg·g^–1^ min^–1/2^)	*C*_2_ (mg·g^–1^)	*R* ^2^
1.07	153.3	0.7688

Figure 4b shows the fit to the intraparticle diffusion (ID) kinetic model in which two stages of adsorption can be determined. As the *q*_t_ versus *t* ^1^/^2^ graph passes close to the origin, it suggests that intraparticle diffusion may play a significant role in the rate-controlling step.^56^ The values of the diffusion constants and intercept are presented in Table 3. The first stage is characterized by the rapid transport of the adsorbate to the adsorbent surface. This is attributed to diffusion through the boundary layer due to the higher diffusion constant (14 mg·g^–1^ min^–1/2^). The second stage has a significantly lower diffusion constant (1.07 mg·g^–1^ min^–1/2^) and a higher intercept (*C*_2_ = 153.3 mg·g^–1^), indicating slower intraparticle diffusion with considerable mass transfer resistance and that equilibrium is reached at this stage. The high value of *C*_2_ may indicate the presence of additional barriers, such as adsorbate accumulation on the outer surface or structural heterogeneity of the adsorbent.^59^ This shows that intraparticle diffusion is not the only mechanism limiting the adsorption kinetics. The data agrees with those of Doğan and Alkan (2009) and Senthilkumaar et al. (2005).^60,61^

### 3.5 Adsorption Mechanism Proposal

The adsorption mechanism of methylene blue (MB) on pecan fruit shells can be elucidated based on the changes observed in the ATR-FTIR spectrum (Figure S2). The main spectral changes indicate the participation of several functional groups present on the lignocellulosic surface of the biosorbent. The decrease in intensity and the shift of the broad band in the 3700–3000 cm^–1^ region suggest the formation of hydrogen bonds between hydroxyl (−OH) groups on the shell surface and the amine groups of MB. In addition, the decrease in intensity in the 1600–1550 cm^–1^ range indicates π–π interactions between the aromatic rings of the dye and the phenolic rings of the lignin present in the material. The changes observed between 1450 and 1000 cm^–1^, attributed to C–N and C–O stretching vibrations, reinforce the hypothesis of additional chemical interactions, such as dipole–dipole and van der Waals (VdW) interactions. Considering the cationic nature of methylene blue and the presence of negatively charged sites on the biosorbent surface, since the experiments were conducted at pH values above pH_PZC_, electrostatic interactions are also plausible. Thus, the adsorption process appears to involve a multifactorial mechanism, including electrostatic interactions, hydrogen bonds, and π–π and VdW interactions (Figure 5), consistent with the Δ*H* values found in this study and with the literature.^24^

**Figure 5:**
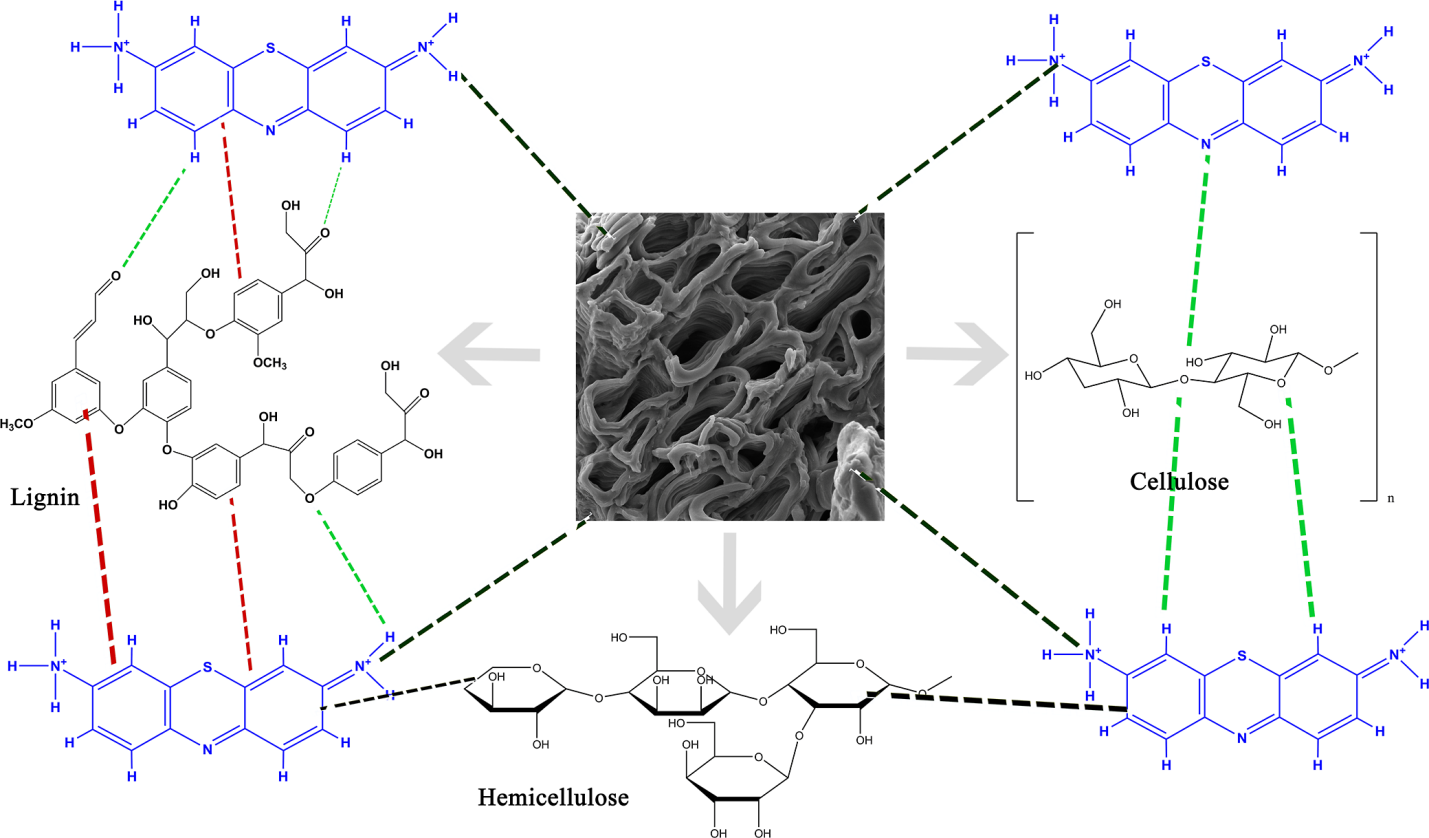
Adsorption mechanism proposed for the adsorption of MB dye onto the PNS biosorbent. The dashed lines indicated the types of interactions: green lines represent H-bonding, red lines show π–π interactions, black lines represent VdW interactions, and brown lines indicate electrostatic interactions.

### 3.6 Comparative Study

Adsorption is influenced by several factors, including the pH and temperature of the solution, the chemical nature of the adsorbate, and the origin and modifications of the adsorbent materials. Many studies have focused on developing materials with a high contaminant adsorption capacity. Methylene blue dye is widely used as a model molecule because it is inexpensive, readily soluble in water, and easily detectable. However, many of these approaches involve complex modification or synthesis steps for the adsorbents, which may result in chemical waste generation and increased production costs that are not always justified by the *q*_m_ values obtained.

Table 4 compares the *q*_m_ values of the PNS biosorbent for MB with those of other modified and unmodified biosorbents. The results indicate that only one other pecan shell-based material has a *q*_m_ value higher than that of PNS. The main advantage of the present study compared with Lima et al., 2019, is associated with the adsorbent preparation route, which is economically favorable and operationally simplified. While the biosorbent proposed by Lima et al., 2019, was produced through a complex procedure involving hydrothermal treatment at 190 °C for 48 h followed by chemical activation with KOH, this approach required considerable energy input, pressurized equipment, and significant reagent consumption; the biosorbent used in this work was derived exclusively from an abundant agricultural residue, in this case, pecan nutshells. Its preparation involved only low-temperature drying (40 °C), grinding, particle size selection, and washing with a diluted NaOH solution, without the need for aggressive activation procedures or severe operating conditions.^24^

**Table 4:** Comparative Study of PNSs with Other Biosorbents^a^

biosorbent	*S*_BET_ (m^2^·g^–1^)	dosage (g·L^–1^)	*Q*_m_ (mg·g^–1^)	[MB] (mg·L^–1^)	isotherm model	ref
activated carbon from bamboo	366	25	83.3	ND	Langmuir	^52^
Algerian Zean oak sawdust	10.17	1	55.82	20	Sips	^46^
yellow passion-fruit waste	30	10	36.96	600	Sips	^48^
Pecan nutshell activated	2342	0.25	1190.62	650	Langmuir	^24^
Tucumã seeds	2.78	7.5	34.41	150	Langmuir	^23^
Metroxylon sagu waste	549.4	5	212.8	300	Langmuir	^45^
soybean hulls	ND	25	169.9	400	Langmuir	^58^
Dacryodes edulis leaf	0.983	10	7.91	100	Langmuir	^25^
watermelon seed hulls	ND	2	26.32	450	Langmuir	^26^
Carya nutshell	0.974	1.5	317.5	500	Sips	this work

a ND: not defined, *S*_BET_: surface area.

In contrast, activated carbon from bamboo requires prolonged pyrolysis and chemical activation steps to achieve high surface areas. Nevertheless, the *q*_m_ values obtained were significantly lower than those of PNS, even when higher dosages were used.^52^ Studies by Lobo et al. (2024) and Amode et al. (2016) investigated the adsorption of MB by biosorbents treated with alkaline solutions. As shown in Table 4, both studies obtained lower adsorption capacity values than PNS, despite using a higher material dosage. Conversely, our material demonstrated an adsorption capacity approximately ten times higher than that of Tucumã seeds.^23,45^

Despite the simplicity of this process, the material showed a good adsorption capacity (317.5 mg·g^–1^ at 25 °C), surpassing several other natural biosorbents reported in the literature.^62–68^ Therefore, this demonstrates that the direct valorization of a residue can yield performance comparable to that of highly engineered activated materials but with significantly lower cost and environmental impact because the preparation cost is minimal (US$0.05 per liter of NaOH solution, considering the Brazilian price of NaOH, US$5 per kilogram of NaOH).

To further ratify the economically viable nature of the proposed process, from an economic perspective, it is possible to conclude, comparatively, that the steps proposed in this work can be classified as sustainable and with a low implementation cost.^68–72^

Considering the main cost components involved in preparing the material, including the acquisition of the raw material, this is practically insignificant due to its residual origin, the consumption of NaOH, and the energy used for drying, grinding, and agitation, as well as the estimated cost per kilogram of the final adsorbent and the operational cost per volume of treated effluent. A direct comparison of this work with the experimental conditions of the studies listed in Table 4 and from Lima et al. (2019),^24^ which requires high energy consumption and a large quantity of reagents, signals the economic advantage of the pecan nutshell-based biosorbent from this work. Therefore, this study shows that the use of a naturally available material processed by simple methods constitutes a sustainable, economically attractive, and technically efficient alternative to the removal of dyes in aqueous systems.

## 4 Conclusion

The present study demonstrated that pecan nutshells (PNSs), a low-cost and sustainable agricultural byproduct, are highly effective biosorbents for the removal of methylene blue dye (MB) from aqueous solutions. The NaOH pretreatment significantly improved the porosity and surface area of the material, thereby favoring exposure of active adsorption sites. The adsorption equilibrium data were best fitted by the Sips isotherm model, which suggests that the biosorbent surface is heterogeneous and has a significant adsorption capacity (317.5 mg·g^–1^ at 25 °C). This value is superior to those of several other natural biosorbents reported in the literature.

Thermodynamic studies revealed that the MB adsorption process onto the PNS is spontaneous and endothermic, with adsorption capacity increasing at higher temperatures. Meanwhile, kinetic analysis indicated that the process follows a pseudo-second-order model, suggesting chemisorption with multiple active sites. The proposed mechanism is predicated on a multifactorial interaction, including electrostatic attractions, hydrogen bonds, π–π stacking, and van der Waals forces, as evidenced by FTIR results.

Furthermore, the study demonstrated that PNS exhibited consistent efficiency across a broad pH range, maintaining its adsorption capacity even under less ideal conditions for cationic dye removal. This finding underscores the material’s robustness and versatility for real-world wastewater treatment applications. Pecan nutshells are notable for their simple preparation, the absence of costly or polluting activation steps, and their excellent adsorption performance compared with other biosorbents.

Due to the low cost, abundance, and biodegradable nature of PNSs, regeneration becomes less economically critical. Furthermore, the management of dye-saturated shells can be accomplished through established biomass recovery methods, such as controlled combustion, coprocessing, or organic recycling. In such processes, thermal or biological processes effectively degrade the adsorbed dye, rendering the spent material environmentally benign. Overall, pecan nutshells have emerged as a promising, environmentally friendly, and scalable solution for the remediation of dye-contaminated effluents. This approach aligns with circular economy and biorefinery principles by adding value to agro-industrial waste while mitigating environmental impacts.

## Supplementary Material



## Notes

van’t Hoff equation plot and FTIR spectra before and after adsorption (PDF)
