# Supplementary material for: Physical and Chemical Characterization, Adsorption Kinetics, Thermodynamic Analysis, and the Mechanism Involved in the Removal of Methylene Blue Dye by a Biosorbent from Pecan Nutshells
Source: ACS Omega. 2026 Jan 22;11(4):6707–17. doi: 10.1021/acsomega.5c12140 (PMC12878767; doi:10.1021/acsomega.5c12140)
Supplement: Supplementary file 1 [file ao5c12140_si_001.pdf]

Physical and chemical characterization, adsorption kinetics, thermodynamic analysis and mechanism involved in Removal of Methylene Blue Dye by a Biosorbent from Pecan Nut Shell

Lucas M. Frescura[†], Rogerio V. Lourega[‡], Nicole W. da Silva[‡], Marcelo B. da Rosa[‡]*.

[†]*Post-Graduate Program of Pharmaceutical Science, Universidade Federal de Santa Maria, Avenue Roraima n 1000, Santa Maria, Rio Grande do Sul, Brazil;*

[‡]*Chemistry Departament, Federal University of Santa Maria, Avenue Roraima n 1000, Santa Maria, Rio Grande do Sul, Brazil.*

*Corresponding author

E-mail address: marcelo.b.rosa@ufsm.br

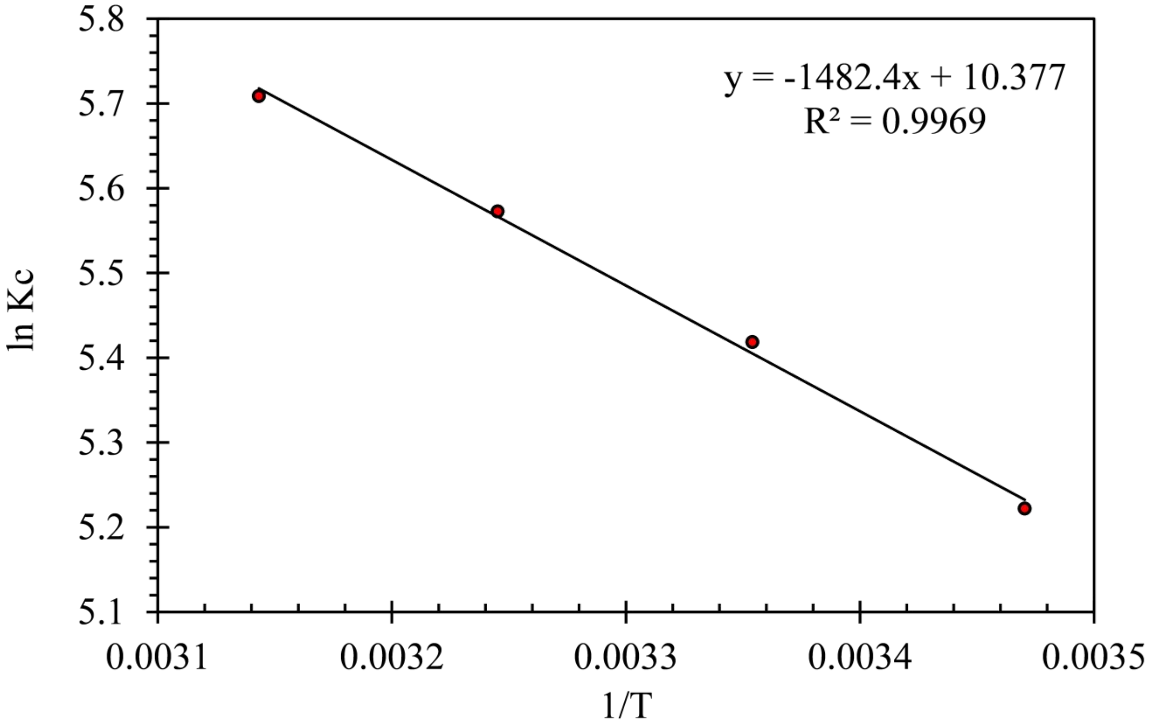

Figure S1. van't Hoff equation plot.

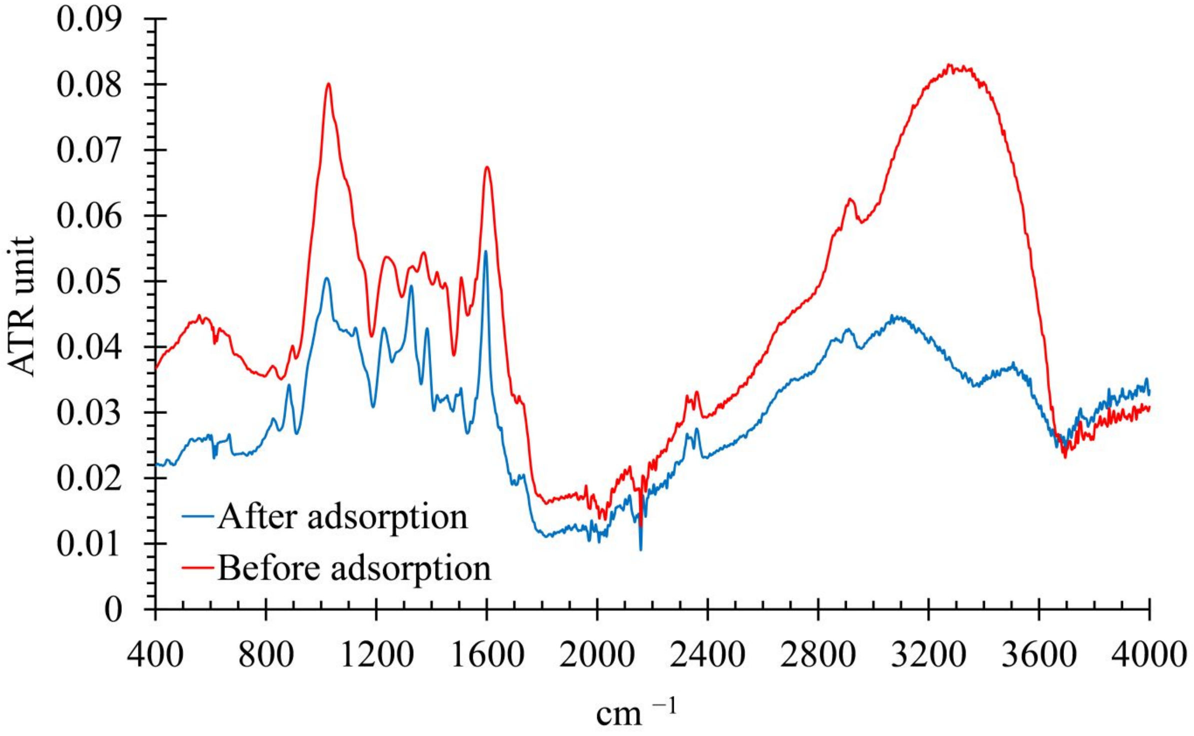

Figure S2. ATR-FTIR spectrum for CNP biosorbent surface before MB sorption (blue line) and after MP sorpion (red line).
